# 

**Published:** 1923-01

**Authors:** 


### GOVERNMENT PUBLICATIONS.

[Copies can be purchased through any bookseller or direct from H.M. Stationery Office, at Imperial House, Kingsway, W.C.2; 28 Abingdon Street, S.W.1; 37 Peter Street, Manchester; 23 Forth Street, Edinburgh; and 1 St. Andrew's Crescent, Cardiff. The prices quoted include postage.]

### Command Paper.

(1761.) Commissioners of Prisons and the Directors of Convict Prisons. Report for 1921-1922, with Appendices. 2s. 1½d.

(1332.) Dairy, England. Milk (Special Designations) Order, Dec. 9, 1922, made by the Minister of Health under Sec. 3 of the Milk and Dairies (Amendment) Act, 1922. 3½d.

### Stationery Office Publications.

Ministry of Health :—

Persons in Receipt of Poor Law Relief in England and Wales. Quarterly Statement, July-September, 1922. 4½d.

Circular 356. Milk and Dairies (Amendment) Act, 1922. Special Designations. To County Councils and Sanitary Authorities (England and Wales). Dec. 12, 1922. 1½d.

Medical Research Council :—

Industrial Fatigue Research Board Reports: XIX. Two Contributions to the Study of Accident Causation. (General Series No. 7.) 1s. 7½d.

Special Report No. 68. Rickets. The Relative Importance of Environment and Diet as Factors of Causation : an investigation in London. 2s. 8d.

Ministry of Agriculture and Fisheries :—

Register of Dairy Cows with Authenticated Milk Records for Year ended October 1, 1921. Vol. V. 2s. 6d.

Statutory Rules and Orders, 1922 :—

National Health Insurance. Central Index Committee Regulations, November 15, 1922. 1½d.

Draft Statutory Rules and Orders, 1922 :—

National Health Insurance, Medical Benefit Consolidated Regulations, 1922. Draft, Nov. 17, 1922, of Regulations proposed to be made by the Minister of Health under the National Health Insurance Acts, 1911 to 1922. 1s. 5½d.

---

### RECENT APPOINTMENTS.

### Public Health.

Dr. A. E. Saunderson, M.R.C.S., L.R.C.P., D.P.H., and Dr. B. Wood-White, M.B., to be Assistant Tuberculosis Officers to the County of East Suffolk.

Dr. S. R. Richardson, M.D., M.B., D.P.H., to be Medical Officer to the Borough of Saffron Walden and Assistant Medical Officer to the County of Essex.

Dr. G. R. Bruce, M.A., M.B, M.D., O.B.E., to be Medical Officer of Health to the Borough of Hastings and St. Leonards.

Dr. P. M. Tolmie, L.R.C.P., L.R.C.S., L.R.F.P.S., to be Assistant Medical Officer of Health to the Hull and Goole Port Sanitary Authority.

Dr. J. Fairley, M.D., D.P.H., to be County Medical Officer for the Isle of Wight.

### Institutional.

Dr. Nancy Martland, M.B., late house surgeon at Oldham Royal Infirmary, to be resident Medical Officer to Fulwood Institution.

Miss G. M. Bulman, to be matron of the Royal Free Hospital, in succession to Miss Cox-Davies, who retires at the end of 1922.

Dr. N. B. Laughton, M.B., Ch.B., D.P.H., to be third Assistant Resident Medical Officer to the County Sanatorium, Harefield, Middlesex.

Dr. Francis Allen, L.R.C.P., M R.C.S., to be Resident Medical Officer at the Borough Sanatorium, Brighton.

### PARLIAMENTARY QUESTIONS AND ANSWERS.

The following answers have been given in the House of Commons by Sir M. Barlow and Major Boyd Carpenter, speaking for the Minister of Health :—

*Housing : Expenditure.*—To Mr. Trevelyan Thomson :

According to a return which has recently been furnished by local authorities who are carrying out housing schemes, the average *all-in* expenditure on houses for which contracts have been made will work out at approximately £1,090 a house. The final settlements of costs which have been made to date relate only to small schemes, covering only 11,000 houses. The average cost of building in these cases, exclusive of land development, professional fees and expenses, works out at £908 a house.

*Housing : Policy.*—To Mr. Trevelyan Thomson (who asked whether, seeing that according to the official returns made by local authorities in 1919, after taking a survey of their housing needs as provided by the Housing Act of 1919, over 800,000 houses were then required to remove existing overcrowding, and that only about 200,000 houses have yet been built, what steps the Government proposes to take to remedy the continued existence of overcrowding) :

My Rt. Hon. Friend can only say that the whole question of future housing policy is receiving his careful consideration.

*Housing : Finance.*—To Colonel Sir Arthur Holbrook :

It is estimated that the commitments under the Assisted Housing Schemes, including slum clearances, will amount to £9,900,000 per annum, of which £9,100,000 per annum will be borne by the Exchequer.

*Poor Law Institutions : Cost of Relief.*—To Mr. Cecil Wilson :

The average weekly cost of relief in general Poor Law institutions, as calculated per head of the inmates (children and adults), was, in the year 1920-21, 30s. 2½d. The comparable amount for children, in separate institutions for children, was 28s. 4¼d. These sums include the cost of accommodation, food, clothing, nursing, education and the salaries, &c., of officers and attendants. Exactly comparable averages for 1913-14 are not available.

The average weekly cost of out-relief as calculated per head of all persons (children and adults) in receipt of it was, in 1913-14, 3s. 0¼d., and in 1920-21, 7s. 4½d. These averages include certain costs of administration.

*Fogs, London.*—To Sir Harry Brittain :

I do not think it is practicable to form any accurate estimate of the financial loss caused by the recent fogs. I will consider the introduction of a Smoke Abatement Bill next session.

*Vaccination.*—To Mr. Frederick Roberts (who asked whether any instruction or advice has been issued by the Ministry of Health in favour of pressure being brought to bear upon persons to submit to vaccination ; and, if so, the authority for this action) :

The answer to the first part of the question is in the negative, and the second part, therefore, does not arise.

*Housing : Statistics.*—To Mr. Trevelyan Thomson :

145,771 houses had been completed by local authorities and public utility societies on November 1, and 39,145 houses had been completed at that date by private builders under the Housing (Additional Powers) Act, 1919, making a total of 184,916 houses completed. In addition 3,056 dwellings have been provided by the conversion of huts and hostels. 30,299 houses remain to be completed, and of these 18,847 were in course of construction on November 1 by local authorities and public utility societies.

*Unemployment : Relief Expenditure.*—To Mr. Daniel Somerville :

The Committee have passed schemes for this winter of a capital cost of £4,709,000, and funds are available for further schemes to bring the total to 12 million pounds.

*Unemployment : Sickness.*—To Sir Walter de Frece :

Some indication of the amount of sickness of all kinds amongst the employed population of the country is furnished by the returns of expenditure by Approved Societies on sickness benefit under the National Health Insurance Acts. From these it appears that there was an increase in the number of weeks for which benefit was paid in 1921, as compared with the previous year of 1.4 per cent. in the case of men, and 8.4 per cent. in the case of women. Many factors must, however, be borne in mind in making a comparison between the figures for the two years and, in particular, the serious epidemic of influenza about the end of the year 1921. The material in the possession of the Ministry does not make it possible to state to what extent the increase may have been attributable to the causes referred to by the hon. member, nor to give comparative figures for different parts of the country.

*Mental Hospitals : Voluntary Boarders.*—To Mr. Robert Richardson (who asked whether the Board of Control have addressed a circular to the medical superintendents of registered hospitals in which it is stated that voluntary boarders in hospitals may leave after giving 24 hours' notice in writing ; and, seeing that according to the statute the hospital superintendent has no such right to demand any notice from the voluntary boarder, would the Minister take steps to see that this statement is at once corrected).

The Circular to which the hon. member refers was addressed to the resident licensees of licensed houses, as well as to the Medical Superintendents of registered hospitals.

Section 229 (6) of the Lunacy Act, 1890, provides for 24 hours' notice in the case of licensed houses, and although the Act is silent as to the notice to be given in the case of registered hospitals, it is in the opinion of the Board of Control reasonable to require the same notice. The Circular suggests that the terms should be made clear to voluntary boarders on admission.

*Housing : Slum Areas.*—To Lieutenant-Commander Kenworthy : The late Government undertook to provide an annual sum not exceeding £200,000 towards the deficits incurred by local authorities in dealing with the clearance of slum areas and the provision of the necessary rehousing. It is the intention of the Government to continue this provision, and a number of local authorities have schemes in progress or in preparation.

*Poor Law Relief : Blind Workers' Dependants.*—To Mr. Groves : It is the policy of the Ministry of Health to secure suitable provision for as many blind persons as possible outside the provisions of the Poor Law.

*Mothers' Pensions.*—Mr. Edwards asked the Chancellor of the Exchequer whether, seeing that he estimates the cost of a practicable scheme of mothers' pensions at £50,000,000 a year, he can state what the present cost is for maintaining those who would be eligible for the above pension ; and whether an opportunity will be given for a discussion on this matter.

Mr. Baldwin : The proportion of mothers at present receiving relief appears to be small, and information with regard to the relief afforded would be of little value. I fear I cannot hold out any hope that it may be possible to provide special facilities for a discussion of the subject.

### LIVINGSTONE COLLEGE.

One of the greatest difficulties that a missionary has to face is that of being sent to a place where there is no medical aid to be obtained for miles. Not only does he himself frequently break down through lack of knowledge of the new conditions, climate, etc., but the natives almost invariably turn to him in times of sickness, merely because he is white. If he cannot help them, they take it that he will not, and ill-feeling is aroused. It is, therefore, most important that a missionary, if he is to be really successful in his work, should have at least an elementary knowledge of medicine, and it was for the purpose of training missionaries in medicine, surgery, and tropical hygiene that Livingstone College was founded in 1893. There were then no schools of tropical medicine and the students in the earlier sessions had the advantage of special teaching in tropical subjects such as few members of the medical profession were able to obtain. Amongst the many distinguished men who have been lecturers at the College was the late Sir Patrick Manson, who has been called the "Father of Tropical Medicine." During the past year students of both sexes from five countries and 21 missionary societies have attended lectures at the College and several enrolled for the short courses on "Care of Health in the Tropics."

Owing to there having been fewer students than usual during the past two years, there is at present a deficit of £586, and the Committee ask those interested in this work to help them to clear this off and to carry out repairs which are urgently needed. The postal address is Livingstone College, Leyton, E.10

